# Level of nutrition knowledge and its association with fat consumption among college students

**DOI:** 10.1186/s12889-016-3728-z

**Published:** 2016-10-04

**Authors:** Najat Yahia, Carrie A. Brown, Melyssa Rapley, Mei Chung

**Affiliations:** 1 Department of Human Environmental Studies, Central Michigan University, Wightman 108, Mt. Pleasant, MI 48859 USA; 2 Jean Mayer USDA Human Nutrition Center on Aging at Tufts University, Boston, MA USA; 3 Department of Public Health and Family Medicine, Tufts University School of Medicine, 136 Harrison Avenue, Jaharis 264, Boston, MA 02111 USA; 4 Department of Biostatistics, Boston University, 801 Massachusetts Avenue, Boston, MA 02118 USA

**Keywords:** Nutrition education, Fat consumption, Nutritional knowledge, University students

## Abstract

**Background:**

Intake of saturated fat, *trans* fat, and cholesterol has been associated with increased risk of coronary heart disease. The aim of this study was to explore whether increased nutrition knowledge is associated with a reduction in the consumption of unhealthy fats in a sample of university students.

**Methods:**

A sample of 231 students, with a mean age of 20 years, was recruited from university campus during spring 2012. Students completed a validated questionnaire related to students’ demographic, nutrition knowledge, and daily fat consumption. Weight, height, and waist circumference were measured. Data were analyzed using one-way ANOVA, chi-square, and student’s *t*-test.

**Results:**

Results indicate that female students have greater nutrition knowledge than male students (the mean nutrition score for women was 5 points higher than that of men (*P* = 0.01)). Nutrition knowledge was negatively correlated with fat and cholesterol intake. Students who consumed more than 35 % calories from fat or >300 mg of cholesterol daily had lower mean nutrition scores than those students with lower fat or cholesterol intake (8 points lower and 7.9 points lower, respectively). Using linear regression for nutrition scores on estimated saturated fat intake and cholesterol intake (controlling for gender, height, weight, age, and dieting), nutrition scores were negatively associated with saturated fat intake (-0.15, *P* <0.0001) and cholesterol intake (-1.38, *P* <0.0001).

**Conclusion:**

Students with greater nutritional knowledge consumed less unhealthy fats and cholesterol. This finding magnifies the role of nutrition education as a potential tool in health campaigns to promote healthy eating patterns among college students. Results of this pilot study can inform the design of future nutrition education intervention studies to assess the efficacy of nutrition knowledge on pattern of fat consumption among college students.

**Electronic supplementary material:**

The online version of this article (doi:10.1186/s12889-016-3728-z) contains supplementary material, which is available to authorized users.

## Background

Cardiovascular disease (CVD) is the leading cause of death in the United States [1, 2]. According to the Centers for Disease Control and Prevention (CDC), about 600,000 American adults die annually of heart disease, or 1 of every 4 deaths [3]. According to a report published by the Michigan Department of Community Health in 2015, the number of cases of heart disease in Michigan is projected to rise from 600,000 to 2.9 million by year 2030 [4]. Recent estimates from the 2013 Behavioral Risk Factor Surveillance System (BRFSS) indicated that 40.6 % of Michigan adults aged 18 years and older reported having high blood cholesterol levels, with higher prevalence among white non-Hispanic adults (42.2 %) than Black, non-Hispanic adults (36.1 %) [5].

A diet high in saturated fat, *trans* fat, and cholesterol is known to raise levels of serum blood cholesterol and can negatively impact cardiovascular health [6]. Accordingly, the American Heart Association recommends that healthy adults should limit their intake of saturated fat to less than 7 % of total daily calories, *trans* fat to less than 1 % of total daily calories, and cholesterol to less than 300 mg a day [7, 8]. Likewise, the 2010 Dietary Guidelines for Americans call for reductions in the consumption of saturated and *trans* fat such as cream, butter, beef fat (tallow, suet), chicken fat, pork fat (lard), stick margarine, and shortening [9].

College is a critical period in life since many lifestyle habits are formed and may persist into adulthood, thereby impacting health [10–13]. Several investigators have reported unhealthy dietary practices among college students such as increasing consumption of fast food and high-fat foods among college students [9–21]. Butler et al. reported significant increases in the percentage of fat and the number of alcoholic beverages consumed daily in a sample of female students at a Midwestern university in the U.S. during their first year of college [20]. Other authors reported a significantly higher intake of total and saturated fat and a lower intake of polyunsaturated and monounsaturated fat in a sample of university students, when compared to the American Heart Association recommendations [21]. The U.S. Surgeon General, in two previous reports, titled “Nutritional Prevention Strategy/Health Eating” and “The Surgeon General's Vision for a Healthy and Fit Nation”, identified universities and schools as sites to raise awareness and educate students about nutrition to help them understand and apply the Dietary Guidelines for Americans recommendations to reduce their risk for heart disease [22, 23].

Literature on the impact of nutrition education on dietary practices has been mixed. Several investigators have reported that nutrition education may change students’ dietary habits and food choices [24–28] while others reported no significant correlation between nutrition knowledge and food choices [29, 30]. Indeed, there could be many factors that impinge on a students’ dietary behaviors, but some basic understanding of nutrition is necessary for a diet change to occur [31]. Whether nutrition education can provide students with the power to select healthier food choices and how *well* students apply food-related knowledge into their daily dietary practices deserve further attention. Thus, this pilot study aimed to explore whether higher nutrition knowledge level is associated with a lower level of fat consumption in a sample of university students from Central Michigan University.

## Methods

### Design and sample

This study was a cross-sectional survey. A sample of 231 students (71 % females and 29 % males), with a mean age of 20 years, was drawn from the university campus. Students were recruited in the classroom (Foods and Nutrition classes) and online (Blackboard announcements) by a Central Michigan University (CMU) Nutrition and Dietetics professor during fall 2011- spring 2012 semesters. The Food and Nutrition classes were introductory nutrition classes offered to all students from any majors and are considered as University Program (UP) - General Education courses. Students who enroll in these classes have no prior academic nutrition knowledge since these classes are introductory nutrition classes offered to all undergraduate students from any majors as part of UP/General Education courses irrespective of students’ year in school. Students agreeing to participate were asked to sign a consent form, in harmony with the Helsinki declaration, and to come to a laboratory classroom for anthropometric measurements and to receive a numerical code for completing a self-administered online questionnaire that included questions related to students’ demographic, nutrition knowledge, and daily fat consumption. The questionnaire was available online via SurveyMonkey Pro (SurveyMonkey.com, LLC, Palo Alto, CA) for about 10 weeks to accommodate students’ response times. Students were not offered any incentives for their participation and were informed that they could withdraw from the study at any time.

Two hundred and fifty students participated in this study, however 19 students were excluded as they did not complete the entire questionnaire. The study protocol was approved by the CMU Institutional Review Board (IRB).

### Data collection

Data were collected in two steps. First, anthropometric parameters, including weight, height, percentage body fat, visceral fat score and waist circumference, were measured by a CMU Nutrition and Dietetics professor.

Weight, percentage body fat, visceral fat, and body mass index were measured using a Tanita bioelectrical impedance analyzer (BIA) SC-331S (Tanita Corporation, Chicago, USA). For BIA measurements, the student’s height, age, and gender were entered into the machine before testing. Then, the student stepped into the BIA’s footpads with bare feet (both feet touched the electrodes). Weight, percentage body fat, visceral fat, and body mass index were recorded from the BIA readings. Since severe dehydration or over-hydration can affect BIA readings, participants’ measurements were taken in the morning after overnight fasting and without consuming alcohol or any stimulant, on an empty bladder, and with no intense exercise. Height was measured while a student was standing erect and without shoes using a stadiometer (Seca 217 Height Measuring Stadiometer, Quick Medical, Issaquah, WA, USA).

Body mass index (BMI) was used to assess students’ body weight category. According to the BMI guidelines published by the National Institutes of Health, participants were categorized into four groups as follows: underweight (BMI ≤18.5), normal weight (18.5 ≤ BMI < 24.9), overweight (25 ≤ BMI < 30), and obese (BMI ≥ 30) [32]. The healthy range for body fat percentage was considered as 8−19 % for males and 17−32 % for females (*Tanita* BIA SC-331S body fat ranges for healthy adults). A visceral fat rating from 1 to 12 was considered healthy for all, and 13 − 59 indicated an excess level of visceral fat. Waist circumference was recorded with a flexible, non-stretchable measuring tape according to the National Institutes of Health guidelines [32].

#### Online questionnaire

In the second step, students were asked to complete an online questionnaire administered via SurveyMonkey (https:// www.surveymonkey.com)*.* Students were told that they could withdraw from the survey at any time and were given an option to skip questions. The questionnaire was pilot-tested on 20 students before it was administered to the students in this study. It consisted of questions related to students’ demographics, dietary fat intake, and nutritional knowledge, and was divided into 3 main parts as follows:

- Part I – *Demographics:* 8 questions related to age, gender, major of study (Science (such as Nutrition and Dietetics, Health and Fitness, Exercise Science, Kinesiology, Community Health) or non-Science major (such as Music, Religion, Theater, Philosophy), year in school, ethnicity, place of residence (on-campus/off-campus), dieting, and smoking status.
- Part II – *Block Dietary Fat Screener*: a validated 17-item food frequency questionnaire (FFQ) was used to assess students’ usual fat intake (Block Fat Screener, NutritionQuest, Berkeley, CA, USA) [33]. A description of the questionnaire is presented in reference 33. The fat screener includes questions about commonly consumed high-fat foods (41 food items) and designed to rank individuals with regard to their usual total fat intake. Data collected from the fat screener were analyzed using prediction equations to generate point estimates of total fat (grams), saturated fat (grams), percent calories from fat, and cholesterol (mg).
- Part III – *Nutrition Knowledge:* 50 questions related to nutritional knowledge. This part was taken from a previously published study by Parmenter and Wardle [34], whose approval was obtained prior to the study. This part includes four sections (dietary recommendations, sources of nutrients, everyday food choices, and diet-disease relationship), each section assessing a different aspect of *general nutrition knowledge* as follows: *section 1*- knowledge of recommendations regarding increasing and decreasing intake of different food groups; *section 2*- nutrient knowledge; *section 3*- food choice; and the last section (*section 4*) about the relationships between diet and disease. The questions were in the form of multiple choice, fill-in-the-blank, and check mark. Each question carries one point for a correct answer. Section 1 (dietary recommendations) has a maximum score of 11; Section 2 (sources of foods/nutrients) has a maximum score of 69; Section 3 (choosing everyday foods) has a maximum score of 10; Section 4 (diet-disease relationships) has a maximum score of 20. Students’ responses to these sections were scored according to the nutrition knowledge questionnaire’s scoring scale [34], and the scores were summed to obtain the nutrition knowledge score. A detailed description of the nutrition knowledge questionnaire and the scoring scale are available at: https://www.ucl.ac.uk/hbrc/resources/resources_eb and described elsewhere [34].

### Data analysis

Power calculation was not performed because this study is exploratory in nature to inform the design of future studies. Statistical analyses were performed using the SAS (9.3, U.S.A) software. Students’ results on the *t*-test and Chi-square test for independence were used to examine differences in the anthropometric characteristics between male and female students. Results are expressed as means ± SD (standard deviation). One-way ANOVA was used to test differences in fat, saturated fat, and cholesterol intake for various demographic variables. Student's *t-*test results were used to examine differences in nutrition score by gender, high fat consumption (>35 % calories), and high cholesterol (>300 mg/day). Separate linear regression for nutrition scores on estimated saturated fat intake and cholesterol intake (controlling for gender, height, weight, age, and self-reported dieting), were used to analyze the association between nutrition scores and saturated fat or cholesterol consumption. Partial correlation coefficients (controlling for gender, height, weight, and age) between nutrition scores and saturated fat intake and cholesterol intake were also calculated. All reported *p* values were based on 2-sided tests with a significance level of 5 % (Additional files 1, 2, 3, 4 and 5.

## Results

### Participants’ characteristics

Two hundred and thirty one students (71 % females and 29 % males), with a mean age of 20.6 ± 2.0 years, participated in this study. The average weight was 69.8 ± 16.7 kg, with an average height of 166.8 ± 9.1 cm. The mean BMI was 24.2 ± 4.4 kg/m^2^. As for percentage body fat, the mean value was 23.9 ± 8.6 %. The mean values of visceral fat scores for males and females were 1.9 ± 1.8 and 3.7 ± 3.4, respectively (Table 1). Based on BMI, the majority of students (68 %) were within the healthy weight category, particularly female students, and about one-third were either overweight (22.5 %) or obese (6.9 %). Of the participating students, 90 % reported as white, and 10 % were African American or other ethnic origin, reflecting the composition of ethnic groups at CMU. Fifty-one percent of students were science majors with a higher percentage for females than males (56 % vs. 37 %, *P* = 0.0088). More than two-thirds of the students (70 %) lived outside of campus, and 80.9 % reported not following any special diet. The percentages of 2nd and 3rd year students were 25 % and 27 %, respectively, while the other 3 levels (1st, 4th, and 5th year) were all under 20 %. The majority of the students were non-smokers (90 %); 5 % were current smokers; and 5 % were former smokers.

**Table 1:** Students’ characteristics

	Male *N* = 67	Female *N* = 164	Total *N* = 231	*P* Value
Age	21.3 ± 2.4	20.3 ± 1.6	20.6 ± 2.0	0.0002
Weight	83.9 ± 16.9	64.0 ± 12.7	69.8 ± 16.7	<.0001
Height	176.7 ± 6.6	162.9 ± 6.5	166.8 ± 9.1	<.0001
Waist circumference	91.1 ± 11.1	81.1 ± 9.6	83.6 ± 11.0	<.0001
BMI	26.0 ± 4.9	23.4 ± 4.0	24.2 ± 4.4	<.0001
% body fat	16.7 ± 8.1	26.8 ± 6.9	23.9 ± 8.6	<.0001
Visceral fat score	3.7 ± 4.2	1.9 ± 1.8	2.4 ± 2.8	<.0001
Ethnicity				0.0905
White	85 %	92 %	90 %	
Black/other	15 %	8 %	10 %	
Body mass index				0.0003
BMI <18.5	3 %	1.7 %	2.6 %	
BMI 18.5− 24.9	47.8 %	76.2 %	68.0 %	
BMI > 25 (overweight)	35.8 %	17.1 %	22.5 %	
BMI > 30 (obese)	13.4 %	4.3 %	6.9 %	
Academic level of study				0.6660
First year	14 %	13 %	14 %	
Second year	20 %	27 %	25 %	
Third year	26 %	27 %	27 %	
Fourth year	20 %	19 %	19 %	
Fifth year	20 %	13 %	15 %	
Major of study				
Health science	37 %	56 %	51 %	0.0078
Non- health science	63 %	44 %	49 %	
Current place of residence				0.6493
On-campus	30.0 %	32.9 %	32.0 %	
Off-campus	70.0 %	67.1 %	68.0 %	
Currently dieting				
No	80.6 %	82.3 %	81.8 %	0.7584
Yes	19.4 %	17.7 %	18.18 %	
Smoking				
Non-smokers	81 %	93 %	90 %	0.0163
Smokers	11 %	4 %	5 %	
Former smokers	9 %	3 %	5 %	

### Daily fat consumption of participating students

Daily mean intake of total fat, saturated fat, and cholesterol based on students’ characteristics is presented in Table 2. Results indicate that there was a significant difference in the consumption of total fat between male and female students. Daily mean intake of total fat was higher among females (92 g/day) compared to males (85.9 g/day) (*P* <0.001). However, saturated fat and cholesterol consumptions were lower for females (23.5 g/day) than males (28.9 g/day).

**Table 2:** Daily mean intake of dietary fats by students’ characteristics

		Total fat (g)	Saturated fat (g)	Cholesterol (g)
Variables				
Gender				
	Males	85.9 ± 24.8	28.9 ± 9.1	298.2 ± 79.3
	Females	92.0 ± 18.8	23.5 ± 6.9	224.5 ± 61.7
	*P*-value*	0.0427	<.0001	<.0001
Body mass index				
	BMI <18.5	81 ± 19.7	22 ± 8.3	222.3 ± 69
	BMI 18.5- 24.9	91.6 ± 20.8	25 ± 7.9	242.1 ± 74.6
	BMI > 25 (overweight)	85.3 ± 19.3	24.6 ± 7.7	248.1 ± 73.9
	BMI > 30 (obese)	95.8 ± 25	29.2 ± 8.7	285.2 ± 79.2
	*P*-value**	0.1120	0.1431	0.1414
Year in school				
	1st-year undergraduate	90.9 ± 24.2	25.3 ± 9.5	250.4 ± 89.5
	2nd -year undergraduate	93.7 ± 21.2	25.9 ± 7.8	250.1 ± 70.6
	3rd -year undergraduate	87.2 ± 18.2	23.9 ± 7.5	236.7 ± 75
	4th -year undergraduate	91.8 ± 20.1	25.7 ± 7.9	249.6 ± 73
	5th -year undergraduate	87.1 ± 23.1	24.6 ± 8	242.8 ± 74.1
	*P*-value**	0.4175	0.6716	0.8536
Major of study				
	Science	85.6 ± 19.7	22.8 ± 6.9	224.1 ± 62.7
	Non-Science	95.1 ± 21.4	27.5 ± 8.4	268 ± 80.5
	*P*-value*	0.0006	<.0001	<.0001
Ethnicity	White	91 ± 20.2	25.2 ± 7.8	242.6 ± 73.6
	Black	83.4 ± 26.4	23.7 ± 9.4	269.8 ± 85.5
	*P*-value*	0.0996	0.3916	0.0996
Residential status				
	On-Campus	90.7 ± 19.7	25.3 ± 7.6	248.5 ± 72.7
	Off-Campus	89.1 ± 23.4	24.5 ± 8.7	240.3 ± 80
	*P*-value*	0.5804	0.4669	0.4381
Dieting	No	91.6 ± 21	25.6 ± 8	250.2 ± 75.4
	Yes	83.8 ± 19.5	22.9 ± 7.4	226.7 ± 70.8
	*P*-value*	0.0277	0.0478	0.0666
Smoking status				
	Non-smoker	90.2 ± 20.5	24.9 ± 7.9	243.2 ± 74.4
	Current smoker	96.6 ± 18.7	29.3 ± 7	285.6 ± 66.8
	Former smoker	83.2 ± 29.3	24.4 ± 10.2	249.3 ± 88.8
	*P*-value**	0.2940	0.1427	0.1409

* *T*-test for independent samples

**1-way ANOVA F-statistic

Regarding the year in school, students’ daily mean intake of total fat did not significantly differ among students, ranging between 87.1 g (lowest) for the 5th year students and 93.7 g (highest) for 3rd year students. Likewise, mean saturated fat intake and cholesterol ranged between 23.9 g and 236.7 g (lowest) for the 3rd year students to 25.9 g and 250.1 g (highest) for the 2nd year students. There was also no significant difference between white and African American students in daily total mean intake of total fat, saturated fat, and cholesterol. Also, students’ residential status and smoking habit were not associated with daily mean intake of total fat, saturated fat, and cholesterol.

On the other hand, students’ daily mean values of total fat intake, saturated fat, and cholesterol were significantly different based on the major of study and dieting. Students majoring in science consumed lower amounts of total fat (85.6 g), saturated fat (22.8 g), and cholesterol (224.1 g) compared to non-science majors (95.1 g, 27.5 g, and 268 g, respectively) (*P* <0.0001). Likewise, students who were on a diet consumed less total fat (*P* <0.0277), saturated fat (*P* <0.0478) and cholesterol compared to students who were not following any diet (Table 2). While the mean cholesterol intake was lower for students on a diet (225.7 g vs 250.2 g), this difference did not achieve statistical significance (*P* = 0.0666).

### Nutrition knowledge survey

**Fig. 1:**
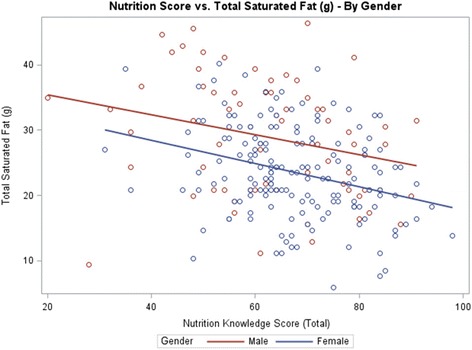
Association of Nutrition Knowledge Score (Total) vs. Total Saturated Fat Intake by Gender

**Fig. 2:**
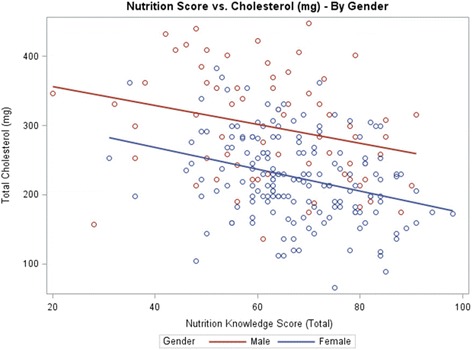
Association of Nutrition Knowledge Score (Total) vs. Cholesterol Intake by Gender

Table 3 shows the results of the students’ responses to the nutrition knowledge survey, indicating that female students scored higher than male students (67.4 ± 12.0 vs. 62.5 ± 15.7, respectively, out of possible 105 points, *P* = 0.01). Students who consumed less than 35 % of daily calories from fat had a higher nutrition score (*P* <0.0001) (Fig. 1) and, similarly, students consuming less than 300 mg of cholesterol had a higher nutrition score (*P* = 0.002) (Table 3) (Fig. 2).

**Table 3:** Mean scores of nutrition knowledge survey

	*n*	Mean NK score	*P*-value*
Total population	231	66.0 ± 13.4	
Gender			
Male	67	62.5 ± 15.7	0.01
Female	164	67.4 ± 12.0	
Percent fat			
<35 %	139	69.8 ± 13.0	<.0001
>35 %	92	61.2 ± 12.4	
Cholesterol			
<300 mg	181	67.7 ± 12.7	0.0002
>300 mg	50	59.8 ± 13.9	

*T-Test for independent Samples

Results of the students’ responses to the four parts of the nutrition knowledge survey showed that students scored highest on the “*Dietary Recommendations*” section compared to the other two sections (*Source of Foods/Nutrients* and *Diet-Disease Relationship*). Female students scored higher than male students on all 3 parts of the nutrition knowledge survey, particularly on the “*Dietary Recommendations*” and the “*Diet-Disease Relationships*” sections, where scores were statistically different between genders (Table 4).

**Table 4:** Mean scores of nutrition knowledge survey and fat screening questionnaire by gender

	Total score	Male	Female	*P* value*
Nutrition knowledge sections (total points)				
Dietary recommendations (11)	8.1 ± 1.7	7.6 ± 2	8.3 ± 1.5	0.002
Sources of foods/nutrients (69)	43.4 ± 8.6	41.8 ± 9.9	44.1 ± 8	0.06
Choosing everyday foods (10)	5.5 ± 1.8	5.2 ± 1.9	5.6 ± 1.8	0.10
Diet-disease relationships (15)	8.9 ± 3.8	7.9 ± 4.5	9.4 ± 3.5	0.01

*T-Test for independent Samples

Controlling for gender, height, weight, age and dieting, there was a significant association between the students’ intake of total fat, saturated fat, and cholesterol and nutrition knowledge score (*P* <0.001) (Table 5).

**Table 5:** Association between fat intake and nutrition knowledge score^a^

	Partial correlation^a^	Regression parameter^b^	*P* value***
Total fat intake (g)	-0.27	-0.42	<.0001
Saturated fat (g)	-0.27	-0.15	<.0001
Cholesterol (mg)	-0.27	-1.38	<.0001

^a^Partial Correlation with Nutrition Score; controlling for gender, height, weight and age

^b^Multiple linear regression of nutrition scores on Total Fat, saturated fat intake or cholesterol intake (controlling for gender, height, weight, age, and self-reported dieting)

***P-value for correlation and regression parameter

## Discussion

The 2010 Dietary Guidelines for Americans recommend that consumption of saturated fat should be limited to <10 % of daily calories, dietary cholesterol to <300 mg per day, and *trans* fat to <1 % of daily calories or as little as possible, primarily to reduce risk of CVD (9). This study looked at the association between nutrition knowledge and fat consumption in a sample of CMU students. Our findings support the general assumption that students’ nutrition knowledge is associated with improved food choices pertaining to types of dietary fats, and the findings concur with results of previous studies [35–37].

In this study, nutrition knowledge was negatively correlated with fat and cholesterol intake. Students who consumed more than 35 % of calories from fat or >300 mg of cholesterol daily had lower mean nutrition scores than those students with lower fat or cholesterol intake (8 points lower and 7.9 points lower, respectively) (*P* <0.001). Using linear regression for nutrition scores on estimated saturated fat intake and cholesterol intake (controlling for gender, height, weight, age, and dieting), nutrition scores were negatively associated with saturated fat intake (-0.15, *P* <0.0001) (Fig. 1) and cholesterol intake (-1.38, *P* <0.0001) (Fig. 2). The results are correlational and therefore cannot show directionality. However, it is likely that nutrition education/ knowledge can lead to improvement of students’ eating habits and healthier food choices. In general, to be able to understand and apply the Dietary Guidelines recommendations for healthful eating practices, some basic understanding of nutrition is necessary [36]. Mazier and McLeod reported that a single course in nutrition was effective at improving the nutrition knowledge of their undergraduate students [37]. A previous study conducted among 269 undergraduate Canadian students to examine the impact of nutrition education on fat consumption found that students who had taken a nutrition course consumed less fat than those with no nutrition education [38]. In this study, students with greater nutrition knowledge consumed lower amounts of total fat, saturated fat, and cholesterol per day compared with students with lower nutrition knowledge scores (Figs. 1 and 2). Proper nutrition knowledge is useful in improving dietary habits, and students are empowered when they have the necessary nutrition knowledge and skills needed to make healthful lifestyle choices [39–41].

Regarding differences in gender, in this study, female students had greater nutrition knowledge than male students (the mean nutrition score for women was 5 points higher than that of men (*P* = 0.01)). This finding was not surprising. In general, women are more likely than men to be interested in diet, nutrition, and body weight, particularly during college years [42–45]. A previous study conducted among 479 Swedish university students found that female students had healthier habits than male students, despite being stressed, whereas male students showed a high level of overweight and obesity and were less interested in nutrition advice and health-enhancing activities [44]. Also, the authors reported that female students were more interested in changing their dietary habits than male students were [44]. Another study evaluating the health knowledge of 428 African American university students found that 75 % of students exhibited high levels of health knowledge, and female students displayed higher levels of knowledge than male students [45]. In our study, female students consumed less saturated fat and cholesterol than male students. This finding is consistent with a previous study among 184 Iranian female students aged 18−35 years showing that female students who perceived themselves as being in the healthy weight range had a significantly lower intake of saturated fat and higher intake of monounsaturated fat compared to other students [46].

The results also showed that students’ daily mean intake of total fat, saturated fat, and cholesterol were significantly different based on major of study and whether they were dieting. Students majoring in science consumed lower amounts of fat compared to non-science majors (*P* <0.0001). Likewise, students who were on a diet consumed less total fat, saturated fat, and cholesterol than students who were not following any diet (*P* <0.0277). It is likely that students majoring in science would have covered more coursework related to nutrition than non-science majors. Also, students following a diet would be more likely to restrict their daily fat intake [42, 47]. Nevertheless, in this study, students’ daily mean intake of total fat, saturated fat, and cholesterol did not differ by body mass index, year-in-school, ethnicity, residential living condition, or smoking status among students. These findings were not surprising [38, 48, 49]. Regarding fat intake, fat intake per se is not the only factor that affect body weight since a clustering of factors including heredity, a low level of physical activity, poor diet, and smoking can contribute to weight gain [50]. Likewise, students’ nutrition knowledge will likely not increase without students taking a nutrition class, irrespective of how long students stay in college. Emrich and Mazier looked at the impact of nutrition knowledge on year-in-school and reported no significant differences in fat intake between first-year students and fourth-year students. However, the authors reported that there were significant differences in fat intake between first-year science students with some nutrition education and those without [38]. This would suggest that nutrition education rather year-in-school is influencing students’ fat intake.

In contrast to our results, which did not reveal any significant differences between on- and off- campus students, Emrich and Mazier reported differences in fat intake between students living on campus and off campus [38]. This could be due to the fact that the residential dining facility at CMU has a program called “Just4U Nutrition”, which offers students all types of healthy and balanced meal plans including vegetarian, vegan, Kosher, low-fat, cholesterol-free, and gluten-free meals. Students also have the option to choose an individualized meal plan that matches their lifestyle. However, our results were in agreement with a previous study conducted among 210 Iranian students to determine factors associated with nutrition knowledge and body weight, which found no significant correlation between nutritional knowledge, body mass index, and smoking status [49].

### Study’s limitations

This study is limited in its small sample size and in that most of the participating students were female (71 % female vs. 29 % male). It is possible that female students may have been more interested in research related to health issues than male students since students voluntarily entered into the study. Nevertheless, the dominance of female participants reflects the university’s student body data and is consistent with the gender composition of previous studies [51, 52]. The limitations of any food frequency questionnaire (FFQ) and FFQ screeners (such as Block Fat Screener) are well recognized [53]. Although Block Fat Screener cannot estimate dietary fat intake accurately, the purpose of this study was not to estimate the amount of dietary fat intake in college students but rather to examine the associations between dietary fat intake level and nutrition knowledge, which is an appropriate use of the FFQ screener in research. Because a FFQ screener is composed of a pre-specified food list or set of behavioral questions, any single screener may not reflect the eating patterns of a given population. Therefore the generalizability of our findings to other populations is limited.

## Conclusion

This pilot study found that students with more nutritional knowledge consumed less unhealthy fats and cholesterol. Students are empowered when they have the necessary knowledge and skills needed to make healthful lifestyle choices [54]. Given the importance of healthy eating in reducing CVD risk factors among students, future research on this topic is needed among this vulnerable age group. Factors such as food cost, food preparation, and cooking methods should also be included in future research since these factors can impact how effectively students can apply nutritional knowledge into their everyday eating habits. In conclusion, the study’s results suggest that students were able to translate nutrition knowledge into their daily diet by reducing their saturated fat and cholesterol intake. This finding magnifies the role of nutrition education as a potential tool in health campaigns to promote healthy eating patterns among college students. We believe that the results of this pilot study can inform the design of future nutrition education intervention studies to assess the efficacy of nutrition knowledge on pattern of fat consumption among college students.

## Additional files

Additional file 1: Consent Form. A sample of the consent form that was given to participants in this study. (TXT 5 kb)
Additional file 2: A copy of the study’s questionnaire. (XML 412 kb)
Additional file 3: A copy of the raw data file. (CSV 157 kb)
Additional file 4: Copy of Regression and Correlation Analysis (CSV 996 bytes)
Additional file 5: SAS data analyses file. (DOCX 19 kb)

## Abbreviations

BRFSS: Behavioral risk factor surveillance system
CDC: Centers for disease control and prevention
CVD: Cardiovascular disease
