# Supplementary material for: Level of nutrition knowledge and its association with fat consumption among college students
Source: BMC Public Health. 2016 Oct 4;16:1047. doi: 10.1186/s12889-016-3728-z (PMC5050673; doi:10.1186/s12889-016-3728-z)
Supplement: Additional file 5: — SAS data analyses file. (DOCX 19 kb) [file 12889_2016_3728_MOESM5_ESM.docx]

***The SAS System***

| ***The MEANS Procedure*** |
| --- |

| **Analysis Variable : Age** | | | | |
| --- | --- | --- | --- | --- |
| **N** | **Mean** | **Std Dev** | **Minimum** | **Maximum** |
| 231 | 20.61 | 1.95 | 18.00 | 32.00 |

***The SAS System***

| ***The FREQ Procedure*** |
| --- |

| **Gender** | | | | |
| --- | --- | --- | --- | --- |
| **f1m0** | **Frequency** | **Percent** | **Cumulative Frequency** | **Cumulative Percent** |
| **Male** | 67 | 29.00 | 67 | 29.00 |
| **Female** | 164 | 71.00 | 231 | 100.00 |

| **Ethnicity** | **Frequency** | **Percent** | **Cumulative Frequency** | **Cumulative Percent** |
| --- | --- | --- | --- | --- |
| **white** | 206 | 89.96 | 206 | 89.96 |
| **non-white** | 23 | 10.04 | 229 | 100.00 |
| **Frequency Missing = 2** | | | | |

| **Major** | **Frequency** | **Percent** | **Cumulative Frequency** | **Cumulative Percent** |
| --- | --- | --- | --- | --- |
| **Health Sciences** | 116 | 50.88 | 116 | 50.88 |
| **Non-Health Sciences** | 112 | 49.12 | 228 | 100.00 |
| **Frequency Missing = 3** | | | | |

| **Year_of_Study** | **Frequency** | **Percent** | **Cumulative Frequency** | **Cumulative Percent** |
| --- | --- | --- | --- | --- |
| **1st year** | 31 | 13.54 | 31 | 13.54 |
| **2nd year** | 58 | 25.33 | 89 | 38.86 |
| **3rd year** | 61 | 26.64 | 150 | 65.50 |
| **4th year** | 44 | 19.21 | 194 | 84.72 |
| **5th year** | 35 | 15.28 | 229 | 100.00 |
| **Frequency Missing = 2** | | | | |

| **housing2** | **Frequency** | **Percent** | **Cumulative Frequency** | **Cumulative Percent** |
| --- | --- | --- | --- | --- |
| **Off Campu** | 157 | 67.97 | 157 | 67.97 |
| **On Campus** | 74 | 32.03 | 231 | 100.00 |

| **smoking** | **Frequency** | **Percent** | **Cumulative Frequency** | **Cumulative Percent** |
| --- | --- | --- | --- | --- |
| **Current smoker** | 13 | 5.63 | 13 | 5.63 |
| **Former smoker** | 11 | 4.76 | 24 | 10.39 |
| **Non smoker** | 207 | 89.61 | 231 | 100.00 |

| **bmi_cat** | **Frequency** | **Percent** | **Cumulative Frequency** | **Cumulative Percent** |
| --- | --- | --- | --- | --- |
| **1** | 6 | 2.60 | 6 | 2.60 |
| **2** | 157 | 67.97 | 163 | 70.56 |
| **3** | 52 | 22.51 | 215 | 93.07 |
| **4** | 16 | 6.93 | 231 | 100.00 |

| **dieting** | **Frequency** | **Percent** | **Cumulative Frequency** | **Cumulative Percent** |
| --- | --- | --- | --- | --- |
| **No** | 189 | 81.82 | 189 | 81.82 |
| **Yes** | 42 | 18.18 | 231 | 100.00 |
